# Prevalence and Prognostic Implications of PD-L1 Expression in Soft Tissue Sarcomas

**DOI:** 10.3389/pore.2021.1609804

**Published:** 2021-07-01

**Authors:** Mohamed Kelany, Thomas FE. Barth, Dina Salem, Marwa M. Shakweer

**Affiliations:** ^1^ Department of Clinical Oncology, Ain Shams University, Cairo, Egypt; ^2^ Department of Pathology, Ulm University, Ulm, Germany; ^3^ Department of Pathology, Ain Shams University, Cairo, Egypt; ^4^ Department of Pathology, Badr University in Cairo (BUC), Cairo, Egypt

**Keywords:** biomarker, prognosis, PD-L1 expression, soft tissue sarcomas, prevalence

## Abstract

**Background:** PD-L1 expression differs from 19 to 92% in various cancer subtypes. Its expression carries a worse prognostic value in various malignancies and could also be used as a predictive marker for immune checkpoint inhibitor response. This study aimed to explore the prevalence of PD-L1 expression in soft tissue sarcomas and the correlation of PD-L1 expression with clinicopathological features.

**Patients and Methods:** The tissue samples of 50 patients with STS were tested for PD-L1 expression using immunohistochemistry (IHC). We followed a 6-step proportional scoring system. The patients were treated at Ain Shams University Hospital from 2011 to 2017. We also explored the correlation of PD-L1 expression with different clinical features of the patients. The chi-square test was used to calculate the differences among variables.

**Results:** Twelve cases (24%) showed positive PD-L1 expression with the highest prevalence in rhabdomyosarcoma and desmoid tumors (2/2 and 2/3 cases, respectively), followed by GIST in 2/4 cases and liposarcoma in 3/11 cases. Patients with positive PD-L1 expression showed a trend for worse survival, with a median overall survival of 11 months vs. 19 months for patients with negative PD-L1 expression (*p*-value = 0.1) and a mean PFS of 6 months vs. 11 months for patients with negative PD-L1 expression (*p*-value = 0.1). However, these findings did not reach statistical significance.

**Conclusion:** Although the results did not reach statistical significance due to the small number of cases, PD-L1 expression could represent a prognostic factor for poor outcome. Larger clinical trials are recommended for the validation of PD-L1 as a poor prognostic biomarker.

## Introduction

Soft-tissue sarcoma is a challenging disease area because it is a heterogeneous disease with over 50 different subtypes (1). Second, it is a rare disease that accounts for over 20% of all pediatric solid malignant cancers and less than 1% of all adult solid malignant cancers (2).

Tumor initiation, progression, and responses to therapy are highly related to the tumor microenvironment, including cells and molecules of the immune system (3,4).

The expression rate of PD-L1 in human malignant tumors has been reported to vary from 19 to 92% and is associated with the progression and poor prognosis of various human cancers. In addition, intratumor infiltration of PD1-positive T-cells was positively correlated with the progression of human malignant tumors (5).

PD-L1 expression is rarely studied in soft tissue sarcoma. In one study that analyzed PD-L1 expression in 82 STS patients, PD-L1 expression was identified in 43% of STS patients and considered an independent adverse prognostic factor for the overall survival of STS patients (6).

We evaluated the expression of PD-L1 in soft tissue sarcoma specimens on paraffin-embedded tissue by immunohistochemistry. Our primary endpoint was the detection of the prevalence of PD-L1 expression in soft tissue sarcoma and its correlation with the clinicopathological features of the tumor. The secondary endpoint was the prognostic significance of PD-L1 in soft tissue sarcoma.

### Sample Size Justification

The necessary sample size was calculated using PASS Sample Size Software, setting the type-1 error (α) at 0.05 (95% confidence interval). The power (1−β) at 0.8 was based on a previous study (7), which showed that PD-L1 expression was 19% in soft tissue sarcoma tumors collectively. The calculation according to these values produced a minimal sample size of 50 cases.

## Methods

This study comprised a total of 50 specimens of surgically removed, formalin-fixed, and paraffin-embedded tumors. Cases were retrieved from the archives of Ain Shams University Hospital during the period from 2011 to 2017 in Cairo, Egypt.

Cases with adequate paraffin tissue samples were included in this study. The study was carried out with full local ethics approval. Various types of soft tissue sarcomas were included. Tumors were classified according to WHO/2013 pathological classification (fourth edition) (8), including desmoid and GIST and excluding bone sarcomas.

We reviewed the medical records for patient data, including age, race, tumor location, histological type and grade, tumor stage based on the seventh edition of the American Joint Committee on Cancer guideline of tumor-node-metastasis (TNM) classification, and resection margin status for resected tumors.

### Immunohistochemical Staining

Immunohistochemistry (IHC) staining was performed on paraffin-embedded tissue sections with a labeled streptavidin-a biotin-peroxidase complex technique using an anti-PD-L1 antibody (CD274 molecule; catalog number: Cell Signaling 13,684, dilution: 1/200). Antigens of PD-L1 were retrieved by microwaving the sections in citrate buffer for 20 min. The final reaction product was developed with diaminobenzidine.

### Immunohistochemical Analysis

We followed a 6-step scoring system (Cologne Score) for the assessment of PD-L1 positivity in sarcomas (Table 1). Only tumor cells with membranous positivity were considered positive for PD-L1. Cytoplasmic positivity was disregarded. Tumor cells were quantified by evaluating the ratio of stained and unstained cells (number of PD-L1-positive tumor cells/number of all tumor cells) (9,10).

**TABLE 1:** Cologne score.

Table of cologne score	Category	0	1	2	3	4	5
	Cut off	<1%	≥1%	≥5%	≥10%	≥25%	≥50%
	Interval	0–1%	≥1%	≥5%	≥10%	≥25%	≥50%
			<5%	<10%	<25%	<50%	<75%

## Results

The cohort consisted of 27 (54%) female and 23 (46%) male patients with a median age of 42.5 years (range 18–79). Most tumors were of grade G2 (34%) and G3 (46%). The majority of the patients did not have metastases at presentation (92%). The most frequent histologic subtypes were liposarcoma (22%), followed by leiomyosarcoma (18%) and ULP (15%). Eighty-four percent (42 patients) underwent surgical treatment, with R0 margins in 38% of these patients. Thirty percent of patients developed metastases, with the lung as the predominant site (see Table 2).

**TABLE 2:** Summary of patient characteristics and clinicopathological features.

Grade	N (%)
FNCLCC:	
G1	6 (12)
G2	17 (34)
G3	23 (46)
GIST:	
Low	3 (6)
High	1 (2)
Surgery	
Yes	42 (84)
No	8 (16)
Margin	
NA	8 (16)
R0	19 (38)
R1	19 (38)
R2	4 (8)
Adjuvant treatment	
Chemotherapy:	
Yes	37 (74)
No	13 (26)
Radiotherapy:	
Yes	28 (56)
No	22 (44)
Recurrence	
Local and nodal	15 (32.6)
Systemic	14 (30.6)
1st Line chemotherapy in systemic recurrence	
Anthracycline:	
Single agent	7 (14)
Combination	15 (30)
TKI: gleevec	1 (2)
Response to the 1st line treatment	
PD	14 (60.9)
PR	6 (26.1)
SD	3 (13)

Patient characteristics	N (%)
Age median (range)	42.5 (18–79)
Sex	
Female	27 (54)
Male	23 (46)
Histology	
Angiosarcoma	1 (2)
Desmoid	3 (6)
Epithelioid	2 (4)
Fibrosarcoma (adu.)	2 (4)
(Myxoid)	4 (8)
GIST	4 (8)
Histiocytic sarcoma	1 (2)
Leiomyosarcoma	9 (18)
Liposarcoma (ULP)	1 (2)
(deDIF.)	1 (2)
(Myxoid)	4 (8)
(ULP)	5 (10)
Pleomorphic sarcoma (UPL)	8 (16)
Rhabdomyosarcoma	2 (4)
Synovial	3 (6)
T Stage	
T1	4 (8)
T2	17 (34)
T3	21 (42)
T4	8 (16)
M Stage	
M0	46 (92)
M1	4 (8)
Original location	
Abdomen/pelvis/R	18 (36)
Extremities	26 (52)
Head and neck	2 (4)
Testicular	1 (2)
Trunk	3 (6)

### PD-L1 Expression in Tumor Specimens and Its Clinical Correlations

**FIGURE 1:**
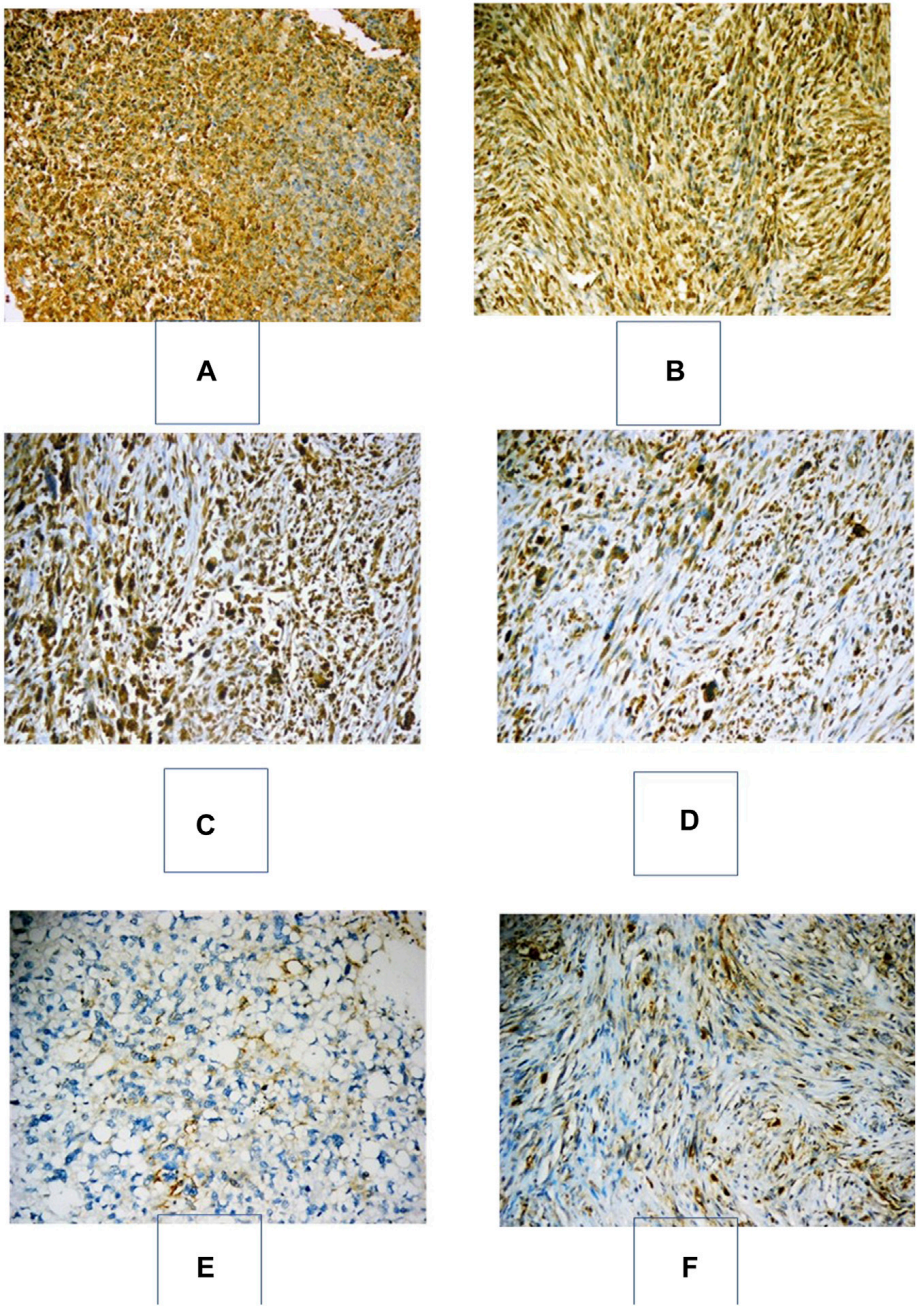
Immunohistochemical expression of PD-L1 in various soft-tissue sarcomas. **(A)**: Embryonal RMS with strong positive expression of PD-L1 in >50% of neoplastic cells, “Cologne Score 5” (PD-L1x200). **(B)** GIST with strong positive expression of PD-L1 in >50% of neoplastic cells, “Cologne Score 5” (PD-L1x200). **(C)** Pleomorphic undifferentiated sarcoma with strong positive expression of PD-L1 in >50% of neoplastic cells, “Cologne Score 5” (PD-L1x200). **(D)** Pleomorphic undifferentiated sarcoma with moderate positive expression of PD-L1 in 25–50% of neoplastic cells, “Cologne Score 4” (PD-L1x200). **(E)**: A pleomorphic liposarcoma with positive membranous moderate immunostaining for PD-L1 in 5–10% of neoplastic cells “Cologne score 2” (PD-L1x200). **(F)**: Fibromatosis with positive membranous moderate immunostaining for PD-L1 in 5–10% of neoplastic cells “Cologne score 2” (PD-L1x200).

**FIGURE 2:**
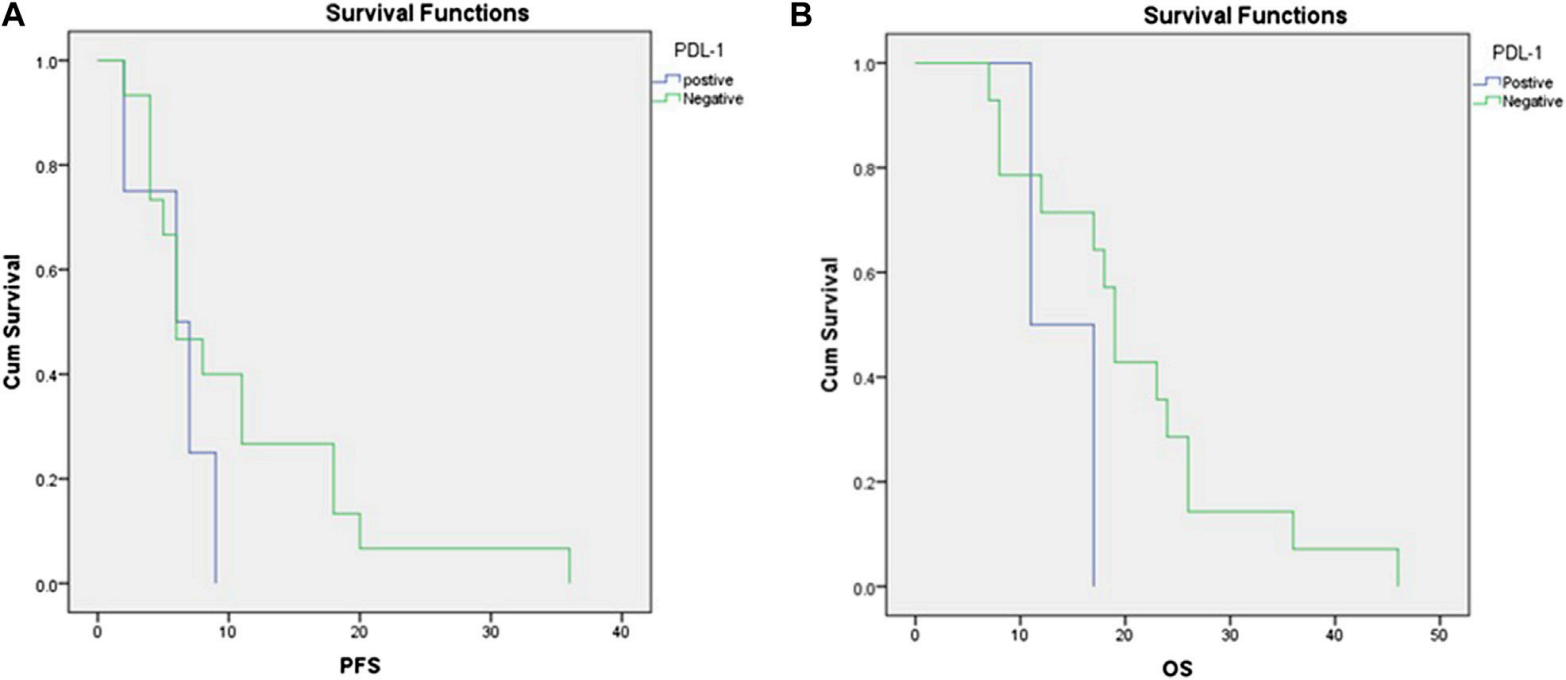
Correlations between PD-L1 expression and **(A)** progression-free survival and **(B)** overall survival.

We defined PD-L1 expression as positive if the Cologne Score equaled three or more, as defined elsewhere (Figure 1; 15). Tumor cell PD-L1 expression was observed in 12 (24%) cases. Of all sarcoma subtypes sampled, the highest prevalence of PD-L1 expression was noted in rhabdomyosarcoma (2/2 cases) and desmoid tumors (2/3 cases), followed by GIST (2/4 cases) and liposarcoma (3/11 cases). The median OS of patients with positive PD-L1 expression (Cologne Score ≥3) was 11 months, while it was 19 months for patients with negative PD-L1 expression [hazard ratio (HR) = 3.086, 95% confidence interval (CI) = 0.59–16.01, *p*-value = 0.14]. The mean PFS of patients with positive PD-L1 expression was 6 months, while it was 10.6 months for patients with negative PD-L1 status [hazard ratio (HR) = 1.806, 95% confidence interval (CI) = 0.55–5.9, *p* value = 0.28] (Figure 2).

## Discussion

In this study, we evaluated the expression of PD-L1 in various sarcoma subtypes and explored its correlations with different clinicopathological features and survival. PD-L1 expression in tumor cells was observed in 12 (24%) of 50 soft tissue sarcoma cases of different subtypes. The tumor cell PD-L1 expression patterns vary in the literature (7), i.e., reports of positive expression in 12% of tumor cells vs. reports that described positive expression in 48% and 65% (11,14). Table 3 summarized the most relevant data published in this area.

**TABLE 3:** Characteristics of the published studies tested the expression of PDL-1 in soft tissue sarcoma.

Study	Year	Pts numbers	Type	Pdl-1 assessment	% Of + expression
(11)	2018	128	Retrospective	IHC	40.4%
(12)	2013	105	Retrospective	IHC	64.7%
(5)	2016	82	Retrospective	IHC	43.%
(7)	2015	47	Retrospective	IHC	8.5%
(13)	2015	59	Retrospective	IHC	59.3%
(14)	2017	163	Retrospective	IHC	11.7%
(15)	2018	46	Retrospective	IHC	45.7%
(16)	2018	81	Retrospective	IHC	59%
(17)	2017	208	Retrospective	IHC	8.65%
(18)	2016	66	Retrospective	IHC	30.3%
(19)	2017	758	Retrospective	RNA seq	41%
(20)	2017	162	Retrospective	RNA seq	21.1%

This discrepancy of the published data may be related to the lack of harmonization of the PD-L1 assay as well as the use of different antibody types and different definitions of a positive result with a specific cut-off. The second reason is related to soft tissue sarcoma itself, as it is not one disease but a heterogeneous group of diseases. We report here a significant variation in PD-L1 expression based on the histological subtypes (*p* = 0.018). This finding was also reported by others, who found that the histological subtypes of soft tissue sarcoma were significantly correlated with PD-L1 expression (*p* = 0.004) (5).

Other than the histological subtypes, we did not identify relationships between tumor PD-L1 expression and clinicopathological characteristics. These results are similar to those of (7) and (5) and in contrast to those of (14), who reported that PD-L1 expression was also significantly associated with higher tumor stage, deep-seated sarcoma, distant metastasis, higher histologic grade, tumor differentiation, and tumor necrosis. Furthermore (21), found that PD-L1 expression was significantly associated with high tumor grade and the age of patients with soft tissue sarcomas.

In other solid malignancies, the level of PD-1/PD-L1 expression is considered an independent factor for worse survival. For example, in renal cell carcinoma, the expression of PD-1 in tumor-infiltrating lymphocytes is considered a poor prognostic factor for survival (21). Additionally, in gastric cancer and lung cancer, PD-L1 expression was established as an independent prognostic factor for poor OS (15,12).

We found that soft tissue sarcoma patients with positive PD-L1 expression showed a median OS of 11 months compared with 19 months for patients with negative PD-L1 expression and a mean PFS of 6 months compared with 11 months for patients with negative PD-L1 expression. We consider this finding clinically significant, as the *p*-value was 0.1 for OS and 0.2 for PFS and therefore indicated a trend. These results confirm the data of (7), who reported a median OS of 10.4 months in PD-L1-positive patients vs. not reached in PD-L1-negative patients, with a *p*-value of 0.8 owing to the small sample size, according to the author’s interpretation.

In contrast (5), found that the expression of PD-L1 in tumor tissue significantly predicted shortened OS [5 years OS rate, 48% vs. 68%; hazard ratio (HR) = 2.545; 95% confidence interval (CI) = 1.16–5.56; *p* = 0.015] (14). reported that soft tissue sarcoma patients with a PD-1+/PD-L1+ phenotype had the shortest survival time and a more progressive STS phenotype. Even in low-stage STS, the five-year survival rate of the PD-1+/PD-L1+ subgroup was only 29%, and the ten-year survival rate was 0%. In contrast, the ten-year survival rates of the PD-1−/PD-L1− subgroups were 94% in low-stage STS and 67% in high-stage STS.

The limitations of our analysis are the heterogeneity of the samples, the small sample size, and the imbalanced histological subtypes between the two groups. The study findings have to be confirmed on a larger scale, involving more patients as well as defined larger groups of each subtype.

Additionally, evaluating PD-L1 expression at an isolated time point or at the interval between biopsy and treatment may not represent its true prevalence. Furthermore, the primary disease and the presence of a metastatic site may further impact the value of PD-L1 expression, and the analysis in our study involved samples mainly originating from early nonmetastatic stages. However, it is well known that this may reflect failed immune surveillance and tumor escape in advanced and metastatic disease.

## Data Availability

The original contributions presented in the study are included in the article/Supplementary Material, further inquiries can be directed to the corresponding author.

## References

[1] Burningham Z Hashibe M Spector L Schiffman JD. The Epidemiology of Sarcoma. Clin Sarcoma Res (2012) 2(1):14. 10.1186/2045-3329-2-14 23036164 PMC3564705

[2] SEER*Stat Databases: November 2017 Submission [Internet]. [cited 2021 Feb 15]. Available from: https://seer.cancer.gov/data-software/documentation/seerstat/nov2017/.

[3] Chen DS Mellman I. Oncology Meets Immunology: The Cancer-Immunity Cycle. Immunity (2013) 39(1):1–10. 10.1016/j.immuni.2013.07.012 23890059

[4] Martin V Raica M Cimpean AM. First-Line Immunophenotyping in the Pathologic Diagnosis of Soft Tissue Tumors. 2004;54(2):122–7.

[5] Kim C Kim EK Jung H Chon HJ Han JW Shin K-H Prognostic Implications of PD-L1 Expression in Patients with Soft Tissue Sarcoma. BMC Cancer (2016) 16(1):434. 10.1186/s12885-016-2451-6 27393385 PMC4938996

[6] Kim C Kim EK Han JW Chon HJ Heo SJ Lee YH Clinical Pattern and Implication of PD-L1 Expression in Soft-Tissue Sarcoma. Jco (2015) 33:10565. 10.1200/jco.2015.33.15_suppl.10565

[7] D'Angelo SP Shoushtari AN Agaram NP Kuk LX Qin L-X Dickson MA Prevalence of Tumor-Infiltrating Lymphocytes and PD-L1 Expression in the Soft Tissue Sarcoma Microenvironment. Hum Pathol (2015) 46(3):357–65. 10.1016/j.humpath.2014.11.001 25540867 PMC5505649

[8] Jo VY Fletcher CDM. WHO Classification of Soft Tissue Tumours: an Update Based on the 2013 (4th) Edition. Pathology (2014) 46(2):95–104. 10.1097/PAT.0000000000000050 24378391

[9] Herbst RS Baas P Kim D-W Felip E Pérez-Gracia JL Han J-Y Pembrolizumab versus Docetaxel for Previously Treated, PD-L1-Positive, Advanced Non-small-cell Lung Cancer (KEYNOTE-010): a Randomised Controlled Trial. Lancet (2016) 387:1540–50. 10.1016/S0140-6736(15)01281-7 26712084

[10] Scheel AH Dietel M Heukamp LC Jöhrens K Kirchner T Reu S Harmonized PD-L1 Immunohistochemistry for Pulmonary Squamous-Cell and Adenocarcinomas. Mod Pathol (2016) 29(10):1165–72. 10.1038/modpathol.2016.117 27389313

[11] Boxberg M Steiger K Lenze U Rechl H von Eisenhart-Rothe R Wörtler K PD-L1 and PD-1 and Characterization of Tumor-Infiltrating Lymphocytes in High Grade Sarcomas of Soft Tissue – Prognostic Implications and Rationale for Immunotherapy. OncoImmunology (2018) 7(3). 10.1080/2162402x.2017.1389366 PMC5790346 29399389

[12] Kim J Moon YJ Kwon KS Bae JS Wagle S Kim KM Tumor Infiltrating PD1-Positive Lymphocytes and the Expression of PD-L1 Predict Poor Prognosis of Soft Tissue Sarcomas. PLoS ONE (2013) 8(12):1–9. 10.1371/journal.pone.0082870 PMC3859621 24349382

[13] Chowdhury F Dunn S Mitchell S Mellows T Ashton-Key M Gray JC. PD-L1 and CD8 ^+^ PD1 ^+^ Lymphocytes Exist as Targets in the Pediatric Tumor Microenvironment for Immunomodulatory Therapy. OncoImmunology (2015) 4(10). 10.1080/2162402x.2015.1029701

[14] Que Y Xiao W Guan Y Liang Y Yan S Chen H PD-L1 Expression Is Associated with FOXP3 + Regulatory T-Cell Infiltration of Soft Tissue Sarcoma and Poor Patient Prognosis. J Cancer 2018;8:2018–25. 10.7150/jca.18683 PMC5559963 28819402

[15] Patel KR Martinez A Stahl JM Logan SJ Perricone AJ Ferris MJ Increase in PD-L1 Expression after Pre-operative Radiotherapy for Soft Tissue Sarcoma. OncoImmunology (2018) 7(7). 10.1080/2162402x.2018.1442168 PMC5993497 29900051

[16] Pollack SM He Q Yearley JH Emerson R Vignali M Zhang Y T-cell Infiltration and Clonality Correlate with Programmed Cell Death Protein 1 and Programmed Death-Ligand 1 Expression in Patients with Soft Tissue Sarcomas. Cancer (2017) 123(17). 10.1002/cncr.30726 PMC5568958 28463396

[17] van Erp AEM Versleijen-Jonkers YMH Hillebrandt-Roeffen MHS van Houdt L Gorris MAJ van Dam LS Expression and Clinical Association of Programmed Cell Death-1, Programmed Death-Ligand-1 and CD8+ Lymphocytes in Primary Sarcomas Is Subtype Dependent. Oncotarget (2017) 8(41). 10.18632/oncotarget.19071 PMC5642642 29050367

[18] Paydas S Bagir EK Deveci MA Gonlusen G. Clinical and Prognostic Significance of PD-1 and PD-L1 Expression in Sarcomas. Med Oncol (2016) 33(8). 10.1007/s12032-016-0807-z 27421997

[19] Bertucci F Finetti P Perrot D Leroux A Collin F le Cesne A PDL1 Expression Is a Poor-Prognosis Factor in Soft-Tissue Sarcomas. OncoImmunology (2017) 6(3). 10.1080/2162402x.2016.1278100 PMC5384364 28405501

[20] Budczies J Mechtersheimer G Denkert C Klauschen F Mughal SS Chudasama P PD-L1 (CD274) Copy Number Gain, Expression, and Immune Cell Infiltration as Candidate Predictors for Response to Immune Checkpoint Inhibitors in Soft-Tissue Sarcoma. OncoImmunology (2017) 6(3). 10.1080/2162402x.2017.1279777 PMC5384369 28405504

[21] Thompson RH Dong H Lohse CM Leibovich BC Blute ML Cheville JC PD-1 Is Expressed by Tumor-Infiltrating Immune Cells and Is Associated with Poor Outcome for Patients with Renal Cell Carcinoma. Clin Cancer Res (2007) 13:1757–61. 10.1158/1078-0432.CCR-06-2599 17363529

